# Potential pathogenic germline variant reporting from tumor comprehensive genomic profiling complements classic approaches to germline testing

**DOI:** 10.1038/s41698-023-00429-1

**Published:** 2023-08-11

**Authors:** Nadine Tung, Kali Chatham Dougherty, Emily Stern Gatof, Kim DeLeonardis, Lauren Hogan, Hanna Tukachinsky, Erica Gornstein, Geoffrey R. Oxnard, Kimberly McGregor, Rachel B. Keller

**Affiliations:** 1 Department of Medical Oncology, Beth Israel Deaconess Medical Center, Boston, MA USA; 2 Foundation Medicine, Inc., Cambridge, MA USA

**Keywords:** Cancer genetics, Cancer genomics

## Abstract

Existing guidance regarding clinically informed germline testing for patients with cancer is effective for evaluation of classic hereditary cancer syndromes and established gene/cancer type associations. However, current screening methods may miss patients with rare, reduced penetrance, or otherwise occult hereditary risk. Secondary finding of suspected germline variants that may confer inherited cancer risk via tumor comprehensive genomic profiling (CGP) has the potential to help address these limitations. However, reporting practices for secondary finding of germline variants are inconsistent, necessitating solutions for transparent and coherent communication of these potentially important findings. A workflow for improved confidence detection and clear reporting of potential pathogenic germline variants (PPGV) in select cancer susceptibility genes (CSG) was applied to a research dataset from real-world clinical tumor CGP of > 125,000 patients with advanced cancer. The presence and patterns of PPGVs identified across tumor types was assessed with a focus on scenarios in which traditional clinical germline evaluation may have been insufficient to capture genetic risk. PPGVs were identified in 9.7% of tumor CGP cases using tissue- and liquid-based assays across a broad range of cancer types, including in a number of “off-tumor” contexts. Overall, PPGVs were identified in a similar proportion of cancers with National Comprehensive Cancer Network (NCCN) recommendations for germline testing regardless of family history (11%) as in all other cancer types (9%). These findings suggest that tumor CGP can serve as a tool that is complementary to traditional germline genetic evaluation in helping to ascertain inherited susceptibility in patients with advanced cancer.

## Introduction

Tumor comprehensive genomic profiling (CGP) is increasingly utilized in the care of patients with advanced cancer. While the primary purpose of tumor CGP is to identify genomic alterations to guide treatment decisions^1^, it can also lead to the detection of potential pathogenic germline variants (PPGV) in cancer susceptibility genes (CSG)^2,3^. PPGV secondary findings can have additional implications for clinical management, including cancer screening and prevention, for both patients and at-risk relatives^4,5^. The American College of Medical Genetics (ACMG) and the European Society of Medical Oncology Precision Medicine Working Group (ESMO PMWG) have emphasized the importance of reporting secondary finding of PPGVs in published guidelines^6,7^. In addition, the American Society of Clinical Oncology (ASCO) and the National Comprehensive Cancer Network (NCCN) both recommend confirmatory germline testing for PPGVs identified via tumor CGP^8,9^.

Recently, expanded guidelines for germline testing based solely on cancer diagnosis have been proffered^10,11^. With the approval of poly (ADP-ribose) polymerase inhibitors (PARPi)^12–16^ for patients with pathogenic germline variants in *BRCA1* and *BRCA2*, the NCCN recommended universal *BRCA1/2* germline testing for patients with advanced ovarian and breast cancer as well as expanded homologous recombination repair (HRR) gene testing for prostate cancer^17–19^. In addition, universal germline testing is now recommended for pancreatic cancer^20^ due to identification of pathogenic germline variants in a high percentage of patients with no family history of cancer who would not have met prior screening criteria for testing^21^. Yet even as broader germline testing considerations are incorporated into national and societal guidelines, the potential for missing clinically actionable germline variants when referral for testing is based on clinical guidelines alone has been recurrently demonstrated through studies in which tumor CGP has led to reporting of previously unrecognized pathogenic germline variants^3,21–24^. Due to the breadth of genes assayed, tumor CGP is particularly poised to identify germline risk in patients with cancer types without established associations with hereditary cancer predisposition syndromes or specific CSGs (i.e., “off-tumor”^6^) in which pathogenic germline variants are nevertheless detected^2,25,26^. We developed a workflow for the identification and reporting of PPGV secondary findings from tumor CGP performed on both tissue and liquid biopsies to maximize the potential of tumor profiling for ascertaining additional opportunities for informed referral for germline testing.

## Results

### Identification of PPGV via tumor CGP

A research dataset of tumor CGP results reported during routine clinical care between January 2021 and June 2022 following launch of the germline banner feature (Supplementary Figure 1A) on Foundation Medicine (FMI) reports was queried to understand the prevalence and actionability of PPGVs detected during tumor profiling. Tumor CGP cases were initially filtered by the presence of short variants (SV) including single nucleotide variants (SNV) and short insertions/deletions (indels) in 24 select CSGs: *ATM, BAP1, BRCA1, BRCA2, BRIP1, CHEK2*, *FH, FLCN, MLH1, MSH2, MSH6, MUTYH*, *PALB2, PMS2, POLE, RAD51C, RAD51D, RET, SDHA, SDHB, SDHC, SDHD, TSC2*, and *VHL*. The decision regarding which CSGs to include in this list was informed by review of published guidelines from the European Society of Medical Oncology Precision Medicine Working Group (ESMO PMWG)^6^ and the American College of Medical Genetics (ACMG)^7^ (Table 1). These 24 CSGs were reported to have a high germline conversion rate (GCR) defined as a > 10% probability of true germline origin when identified through tumor-only sequencing^6^. (Notably, analysis of an expanded case series informed recent updates to ESMO PMWG guidance which utilized a > 5% GCR threshold and yielded a more extensive CSG list including *BARD1*, *CDKN2A*, *DICER1*, *POLD1, PTCH1*, *SMAD3*, *SMARCA4*, *SMARCB1*, and *SUFU* in certain contexts^27^). While *ATM* and *CHEK2* were excluded from the ESMO PMWG (2019) and ACMG guidelines due to low penetrance/moderate risk, they were retained in the selected gene list because germline variants in these genes have been associated with poor survival in breast cancer^28^ and patients harboring these variants have been shown to benefit from screening^29^. A recent update to the ESMO PMWG guidance now includes *ATM* and *CHEK2* due to common inclusion for clinical germline testing^27^. A few highly penetrant CSGs (*APC, NF1, RB1*, and *TP53*) were not selected for inclusion due to low GCR in adults; while conversion rates for variants in these genes are high in patients age < 30 years^6^, this represents a minority of the FMI testing population. However, mention of the germline potential of known or suspected pathogenic germline variants in these 4 CSGs, as well as in 8 additional CSGs excluded for other reasons (*MEN1*, *NF2*, *PTEN, SMAD4, STK11, TGFBR2, TSC1*, and *WT1*), is discussed in ‘Potential Germline Implications’ in the Genomic Findings section of the FMI report, (Table 1, Supplementary Fig. 1B). Although both ESMO PMWG and ACMG guidelines recommend reporting of suspected germline *MUTYH* as a secondary finding only when two pathogenic/likely pathogenic variants are present^6,7^, due to the dependence of clinical guidelines for *MUTYH* carriers on family history^11^, it was decided to highlight both monoallelic and biallelic *MUTYH* variants to allow clinical interpretation to be at the discretion of the ordering provider.

**Table 1:** Foundation Medicine Reported & ESMO/ACMG Recommended Reported Cancer Susceptibility Genes (CSG).

Cancer Susceptibility Gene (CSG)	Associated Disease/Phenotype	Foundation Medicine		ESMO PMWG (Mandelker et al. 2019)	ACMG (Miller et al. 2021)	Exclusion Reason(s)
		Germline Banner	Report			
*APC*	Familial Adenomatous Polyposis (FAP)		**X**	**X**^**2**^	**X**	FMI Banner: C^**2**^
*ATM*	Ataxia-Telangiectasia (AT); Breast, Pancreatic Cancer Susceptibility	**X**	**X**			ESMO: P, A ACMG: P, A
*BAP1*	Tumor Predisposition Syndrome 1	**X**	**X**	**X**^**1**^		ACMG: A
*BMPR1A*	Juvenile Polyposis Syndrome (JPS)				**X**	FMI Banner/Report: NC ESMO: C
*BRCA1*	Hereditary Breast & Ovarian Cancer	**X**	**X**	**X**	**X**	
*BRCA2*	Hereditary Breast & Ovarian Cancer	**X**	**X**	**X**	**X**	
*BRIP1*	Ovarian, Breast Cancer Susceptibility	**X**	**X**	**X**		ACMG: P, A
*CHEK2*	Breast, Colorectal, Prostate Cancer Susceptibility	**X**	**X**			ESMO: P ACMG: P
*FH*	Hereditary Leiomyomatosis & Renal Cell Cancer	**X**	**X**	**X**^**1**^		ACMG: NP
*FLCN*	Birt-Hogg-Dube Syndrome (BHD)	**X**	**X**	**X**^**1**^		ACMG: NP
*MAX*	Hereditary Paraganglioma-Pheochromocytoma Syndrome				**X**	FMI Banner/Report: NC ESMO: NP
*MEN1*	Multiple Endocrine Neoplasia Type I		**X**		**X**	FMI Banner: C ESMO: C
*MLH1*	Lynch Syndrome	**X**	**X**	**X**	**X**	
*MSH2*	Lynch Syndrome	**X**	**X**	**X**	**X**	
*MSH6*	Lynch Syndrome	**X**	**X**	**X**	**X**	
*MUTYH*	*MUTYH*-Associated Polyposis (MAP)	**X**	**X**	**X**	**X**	
*NF1*	Neurofibromatosis Type 1		**X**	**X**^1,2^		FMI Banner: C^**2**^ ACMG: NP
*NF2*	Neurofibromatosis Type 2		**X**		**X**	FMI Banner: C ESMO: C
*PALB2*	Breast, Pancreatic Cancer Susceptibility	**X**	**X**	**X**	**X**	
*PMS2*	Lynch Syndrome	**X**	**X**	**X**	**X**	
*POLE*	Colorectal Cancer Susceptibility	**X**	**X**	**X**^**1**^		ACMG: A
*PTEN*	PTEN Hamartoma Tumor Syndrome		**X**		**X**	FMI Banner: C ESMO: C
*RAD51C*	Breast, Ovarian Cancer Susceptibility	**X**	**X**	**X**		ACMG: P, A
*RAD51D*	Breast/Ovarian Cancer Susceptibility	**X**	**X**	**X**		ACMG: P, A
*RB1*	Retinoblastoma		**X**	**X**^**2**^	**X**	FMI Banner: C^**2**^
*RET*	Familial Medullary Thyroid Cancer; Multiple Endocrine Neoplasia Type 2A/2B	**X**	**X**	**X**	**X**	
*SDHA*	Hereditary Paraganglioma-Pheochromocytoma Syndrome	**X**	**X**	**X**		ACMG: T
*SDHAF2*	Hereditary Paraganglioma-Pheochromocytoma Syndrome			**X**	**X**	FMI Banner/Report: NC
*SDHB*	Hereditary Paraganglioma-Pheochromocytoma Syndrome; GIST	**X**	**X**	**X**	**X**	
*SDHC*	Hereditary Paraganglioma-Pheochromocytoma Syndrome; GIST	**X**	**X**	**X**	**X**	
*SDHD*	Hereditary Paraganglioma-Pheochromocytoma Syndrome	**X**	**X**	**X**	**X**	
*SMAD4*	Juvenile Polyposis Syndrome (JPS)		**X**		**X**	FMI Banner: C ESMO: C
*STK11*	Peutz-Jeghers Syndrome		**X**		**X**	FMI Banner: C ESMO: C
*TGFBR2*	Hereditary Non-Polyposis Colorectal Cancer (HNPCC)		**X**			FMI Banner: C ESMO: C ACMG: NP
*TMEM127*	Hereditary Paraganglioma-Pheochromocytoma Syndrome				**X**	FMI Banner/Report: NC ESMO: C
*TP53*	Li-Fraumeni Syndrome		**X**	**X**^1,2^	**X**	FMI Banner: C^1,2^
*TSC1*	Tuberous Sclerosis Complex		**X**		**X**	FMI Banner: C ESMO: C
*TSC2*	Tuberous Sclerosis Complex	**X**	**X**	**X**	**X**	
*VHL*	von Hippel-Lindau Syndrome	**X**	**X**	**X**^**1**^	**X**	
*WT1*	*WT1*-Related Wilms Tumor		**X**		**X**	FMI Banner: C ESMO: C

^1^Tumor Type Restricted ^2^Age Restricted To < 30 Years Exclusion Reasons Key*:* *A* Unclear Actionability/Lack Of Consensus Regarding Clinical Management, *C* < 10% Germline Conversion Rate (GCR), *P* Low Penetrance, *NC* Not Covered On FMI Assays, *NP* No Reason Provided, *T* Technical Concerns Regarding Detection. *ACMG* American College Of Medical Genetics, *FMI* Foundation Medicine, Inc., *ESMO-PMWG* European Society Of Medical Oncology Precision Medicine Working Group.

Next, SVs in these 24 CSGs were filtered using variant allele frequency (VAF) thresholds. The respective thresholds for tissue CGP ( > 10% VAF) and liquid CGP ( > 30% VAF) were internally developed and validated to capture > 95% (range 96.6-100%) of exemplar germline variants (Supplementary Fig. 2). Lastly, qualifying variants were filtered based on classification in ClinVar^30^ (accessed March 1st, 2022). Variants classified as ‘Pathogenic’, ‘Pathogenic/Likely Pathogenic’, or ‘Likely Pathogenic’ (P/LP) in ClinVar by more than one submitter or by an expert panel were retained. If variants were not registered in ClinVar, they were filtered out. Because germline and somatic pathogenicity are differentially defined, this sometimes resulted in variants being excluded from the germline banner while still reported as pathogenic or likely pathogenic on tumor CGP results. Variants that met all three filtering criteria were considered Potential Pathogenic Germline Variants (PPGV). Any sample harboring ≥ 1 PPGV was included in the PPGV+ cohort. FMI tumor CGP reports for these samples feature a germline banner to highlight the identification of a PPGV and encourage pursuit of dedicated germline testing for confirmation (Supplementary Fig. 1A). After filtering, the PPGV+ cohort consisted of 12,176 unique patients who underwent tumor CGP (10,437 with tissue CGP and 1739 with liquid CGP).

### PPGV pan-cancer landscape

**Fig. 1:**
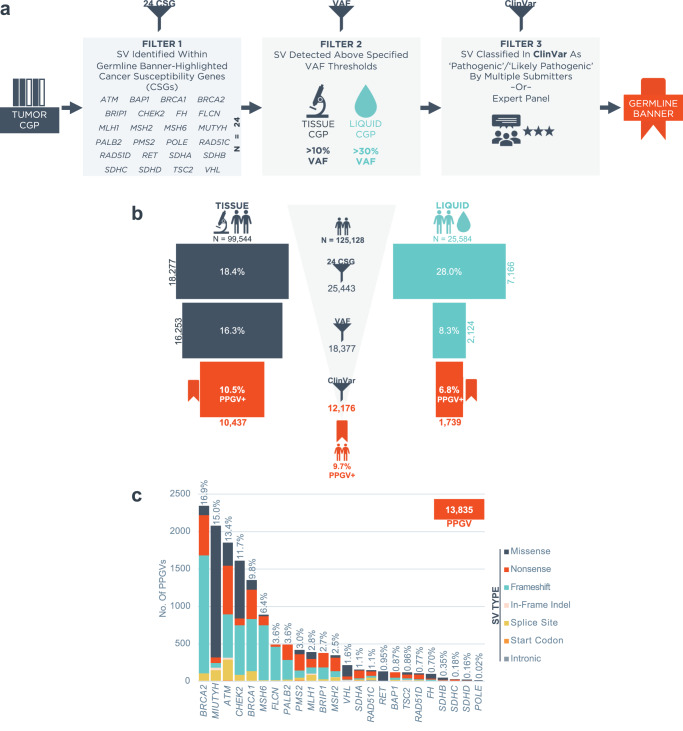
Identification of potential pathogenic germline variants (PPGV) via tumor comprehensive genomic profiling (CGP). **a** Criteria for filtering of PPGVs and inclusion of the germline banner on Foundation Medicine reports. **b** Identification of the PPGV+ cohort (*Middle*) by applying filtering criteria to tissue CGP (*Left*) and liquid CGP (*Right*). The % and count of samples after each filtering step are indicated. **c** Pan-cancer distribution of PPGVs across the 24 germline banner CSGs. *P/LP* Pathogenic/Likely Pathogenic, *SV* Short Variant (incl. single nucleotide variants [SNV] and short insertions/deletions [indels]), *VAF* Variant Allele Frequency.

Analysis was performed on a pan-cancer cohort of 125,128 tumor samples assayed using either tissue- (*n* = 99,544) or liquid-based (*n* = 25,584) CGP. A detailed breakdown of cancer types assayed is presented in Supplementary Fig. 3. Notably, liquid CGP was enriched for lung and prostate cancers while tissue CGP was enriched for colorectal (CRC) and ovarian cancers. Using the filtering scheme inclusive of a selective CSG list, VAF, and pathogenicity based on ClinVar (Fig. 1a), 9.7% of cases were found to harbor ≥ 1 PPGV (Fig. 1b). The most common genes in which PPGVs were identified pan-cancer were *BRCA2* (16.9% of PPGVs), *MUTYH* (15.0%)*, ATM* (13.4%)*, CHEK2* (11.7%), and *BRCA1* (9.8%) (Fig. 1c).

Multiple PPGVs were identified in 10% of PPGV+ cases (*n* = 1266) and these cases were enriched in tissue CGP (1.2% versus 0.2% of liquid CGP) (Supplementary Fig. 4A, B) as expected due to the less stringent VAF threshold applied to tissue cases. It was hypothesized that tumors with microsatellite instability (MSI-H) and high tumor mutational burden (TMB-H; ≥ 10 Mut/Mb) might explain these multi-PPGV cases wherein an excess of passenger mutations might lead to a higher probability of mutations passing our set of filters. Indeed, MSI-H tumors accounted for 53% of multi-PPGV tissue CGP cases and non-MSI-H/TMB-H tumors accounted for another 17% (Supplementary Fig. 4C). The cancer types with the highest percentages of multi-PPGV tissue CGP cases (i.e., uterine, CRC, skin non-melanoma, and GI-other) were explained by MSI-H/TMB-H tumors in most instances (70–100%; Supplementary Fig. 4D).

### Clinicogenomic features of the PPGV+ cohort

The clinicogenomic characteristics of the PPGV+ and PPGV- cohorts were compared (Table 2). PPGV+ patients tended to be younger at time of biopsy (median 65 versus 67 years, *p* < 0.001) and the percentage of young-onset patients ( ≤ 50 years) was higher in the PPGV+ cohort (13.8% versus 11.5%, *p* < 0.001). Female sex was more common in the PPGV+ population (55.3% versus 51.5%, *p* < 0.001), potentially biased by the high frequency of breast cancers in the patient population studied. MSI-H (11.8% versus 1.0%, *p* < 0.001) and TMB ≥ 10 Mut/Mb in tissue CGP cases (30.7% versus 15.2%, *p* < 0.001) were each enriched in the PPGV+ cohort, which could indicate that some PPGVs identified in this subset of tumors may be somatic passenger mutations. Genomic ancestry was also different between the cohorts with European (79.2% versus 74.6%) ancestry overrepresented and African (9.3% versus 12.0%) and Admixed American (7.8% versus 9.0%) ancestry underrepresented in the PPGV+ population (*p* < 0.001) which may reflect the underrepresentation of non-European populations in the ClinVar database^31–34^.

**Table 2:** Clinicogenomic Characteristics Of The CGP Cohort.

Characteristic	All Cases *N* = 125,128		PPGV+ Cases *N* = 12,176		PPGV- Cases *N* = 112,952		*P*-Value (FDR)
	*n*	%	*n*	%	*n*	%	
**Age At Bx, Median (Q1, Q3)**	66 (58, 74)		65 (57, 73)		67 (58, 74)		< 0.001
≤ 50 Years	14,452	11.8%	1652	13.8%	12,800	11.5%	< 0.001
Unknown	2313	-	212	-	2101	-	-
**Sex**							< 0.001
Female	64,885	51.9%	6730	55.3%	58,155	51.5%	-
Male	60,243	48.1%	5446	44.7%	54,797	48.5%	-
**Predicted Ancestry**							< 0.001
European	93,844	75.1%	9633	79.2%	84,211	74.6%	‡
African	14,646	11.7%	1126	9.3%	13,520	12.0%	‡
Admixed American	11,132	8.9%	943	7.8%	10,189	9.0%	‡
East Asian	4148	3.3%	360	3.0%	3788	3.4%	-
South Asian	1214	1.0%	98	0.8%	1116	1.0%	-
Unknown	144	-	16	-	128	-	-
**Tumor Bx Site**							< 0.001
Local	47,643	43.6%	4860	46.2%	42,783	43.3%	‡
Metastatic	27,103	24.8%	2976	28.3%	24,127	24.4%	‡
Lymph Node	8941	8.2%	935	8.9%	8006	8.1%	‡
Peripheral Blood	25,584	23.4%	1739	16.5%	23,845	24.1%	‡
Unspecified	15,857	-	1666	-	14,191	-	-
MSI-H	2562	2.0%	1432	11.8%	1130	1.0%	< 0.001
TMB ≥ 10 Mut/Mb^1^	16,709	16.8%	3200	30.7%	13,509	15.2%	< 0.001
bTMB ≥ 10 Mut/Mb^2^	2832	16.5%	215	18.3%	2617	16.4%	0.09

^1^Tissue CGP Only ^2^Liquid CGP Only w/ ctDNA Tumor Fraction (cTF) ≥ 1% A double dagger (‡) indicates a Significant Subcategory Difference. Statistical analysis was performed using Fisher’s Exact Tests or Chi-Squared Tests, as appropriate, and the p.adjust function in R was used for *p*-value multiple hypothesis corrections. *bTMB* Blood Tumor Mutational Burden, *CGP* Comprehensive Genomic Profiling, *FDR* False Discovery Rate, *MSI-H* Microsatellite Instability-High, *PPGV* Potential Pathogenic Germline Variant, *TMB* Tumor Mutational Burden.

Due to the possible limitations of relying on ClinVar to identify PPGVs in the cohort, the SV in germline banner CSGs that had been filtered out due to insufficient evidence in ClinVar (i.e., either unregistered variants or variants classified as P/LP by a single submitter) were explored. This analysis was restricted to liquid CGP due to the higher stringency of the liquid VAF threshold which leads to a lower rate of false positive PPGV calls from the VAF filtering step. ClinVar-filtered variants were present in 22 of 24 germline banner CSGs and most were either variants of uncertain significance (VUS; 46.6%) or were absent from ClinVar (44.8%) (Supplementary Fig. 5A). Fifteen variants in *BRCA1* and 28 variants in *BRCA2* were filtered out due to insufficient evidence in ClinVar, with a high fraction (47% and 71%, respectively) identified on-tumor (i.e., in breast/ovarian/pancreatic/prostate cancers), as expected for true pathogenic germline variants (Supplementary Fig. 5B). Of the predicted pathogenic (i.e., loss-of-function) *BRCA1/2* variants in ovarian cancer that had been discarded (*n* = 2), one variant is characterized as ‘Pathogenic’ by a single submitter (*BRCA1* S324fs*17; ClinVar Variation ID: 946287), while the other is absent from the database (*BRCA2* I979fs*12). Notably, both patients harboring these variants were predicted to be of non-European ancestry. Overall, SV filtered out due to ClinVar were more frequently found in patients predicted to be of African (31.8%, *p* < 0.001), South Asian (26.1%, *p* = 0.42), East Asian (24.4%, *p* = 0.38), and Admixed American (20.8%, *p* = 0.42) descent than in patients predicted to be of European descent (17.2%) (Supplementary Fig. 5C).

### Clinical impact of PPGV secondary findings from tumor CGP

**Fig. 2:**
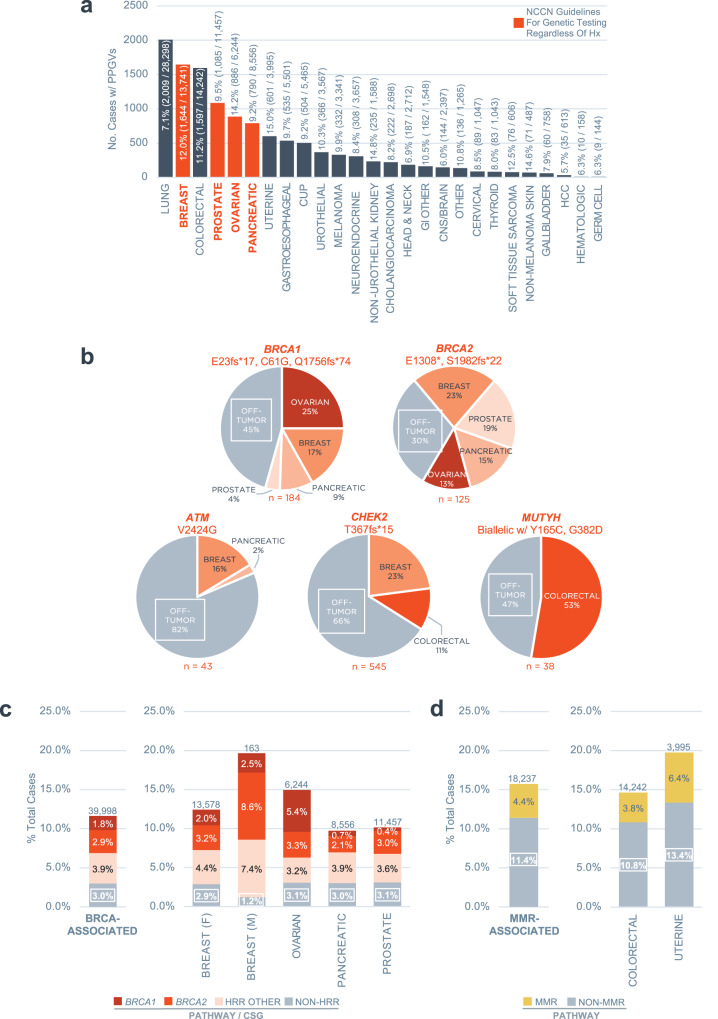
Clinical impact of secondary finding of PPGVs on tumor CGP. **a** PPGVs detected across cancer types via combined tissue and liquid CGP. The prevalence and count of total PPGV+ cases for each cancer type are shown. The cancer types with broad recommendations for germline testing in the NCCN guidelines regardless of family and personal history are colored red, i.e., germline testing is recommended universally in ovarian and pancreatic cancer, universal *BRCA1/2* testing is recommended in metastatic HER2-negative breast cancer, and testing is recommended for all patients with metastatic/advanced/high-risk localized prostate cancer. **b** Distribution of cancer types in which select founder mutation PPGVs were detected in *BRCA1* (E23fs*17, C61G, Q1756fs*74), *BRCA2* (1308*, S1982fs*2), *ATM* (V2424G), *CHEK2* (T367fs*15), and *MUTYH* (biallelic involving Y165C and/or G382D). **c** PPGVs identified in *BRCA*-associated cancers (Breast/Ovarian/Pancreatic/Prostate). HRR Other Genes include *ATM, BAP1, BRIP1, CHEK2, PALB2, RAD51C, RAD51D*. Non-HRR Genes include *FH, FLCN, MLH1, MSH2, MSH6, MUTYH, PMS2, POLE, RET, SDHA, SDHB, SDHC, SDHD, TSC2, VHL*. The total number of cases (N) for each cancer type is indicated above the bar. **d** PPGVs identified in Lynch Syndrome-associated cancers (Uterine/Colorectal). MMR Genes include *MLH1, MSH2, MSH6, PMS2*. Non-MMR Genes include *ATM, BAP1, BRCA1, BRCA2, BRIP1, CHEK2, FH, FLCN, MUTYH, PALB2, POLE, RAD51C, RAD51D, RET, SDHA, SDHB, SDHC, SDHD, TSC2, VHL. C2, VHL*. The total number of cases (N) for each cancer type is indicated above the bar. *CSG* Cancer Susceptibility Gene, *HRR* Homologous Recombination Repair, *MMR* Mismatch Repair, *NCCN* National Comprehensive Cancer Network, *PPGV* Potential Pathogenic Germline Variant.

As expected, cancer types with broad recommendations for germline testing in the NCCN guidelines (ovarian, pancreatic, breast, and prostate) exhibited both high numbers and high frequencies of PPGV+ cases (Fig. 2a). Appropriate phenotypic clustering of PPGVs in specific CSGs was observed, e.g., *VHL* in kidney (7.7%), *BRCA1/2* in breast (2.0% and 3.2%, respectively) and ovarian (5.4% and 3.3%), *BRCA2* in prostate (3.0%), and mismatch repair pathway (MMR) genes in uterine (*MSH2* 0.9%, *MSH6* 3.9%, *PMS2* 0.8%, *MLH1* 0.9%) and colorectal (*MSH2* 0.6%, *MSH6* 1.8%, *PMS2* 0.5%, *MLH1* 0.8%) cancers (Supplementary Fig. 6). Notably, a significant proportion of PPGVs were identified in cancer types for which germline testing guidance is limited or for which universal testing is infeasible due to low pathogenic germline variant prevalence. This could impact a substantial population of patients if these variants were confirmed to be germline (Fig. 2a). For example, PPGVs were identified in 7.1% of patients with lung cancer which represents >2000 patients in this cohort for whom germline referral and testing could be considered.

Known pathogenic founder mutations were identified in a high percentage of tumor types not typically associated with these inherited variants (Fig. 2b, Supplementary Fig. 7): 45% of select *BRCA1* (E23fs*17, C61G, Q1756fs*74) and 30% of select *BRCA2* founder mutations (E1308*, S1982fs*22) were identified outside of breast/ovarian/pancreatic/prostate cancer; 82% of select *ATM* founder mutations (V2424G) were identified outside of breast/pancreatic cancer; 66% of select *CHEK2* founder mutations (T367fs*15) were identified outside of breast/colorectal cancer; and 47% of select *MUTYH* founder mutations – either homozygous Y165C/G382D (VAF ≥ 99%) or compound heterozygous involving Y165C/G382D (i.e., Y165C + G382D or Y165C/G382D in combination with another *MUTYH* PPGV) – were identified outside of CRC.

Lastly, PPGVs identified in *BRCA1/2*, and other homologous recombination repair (HRR) pathway CSGs in *BRCA*-associated cancers (Fig. 2c) as well as MMR pathway CSGs (*MLH1, MSH2, MSH6, PMS2*) in Lynch Syndrome-associated cancers (Fig. 2d) were examined. Alternate (i.e., non-*BRCA*) HRR pathway genes constituted 3.9% of PPGVs identified in *BRCA*-associated cancers. Moreover, 3.0% of PPGVs in *BRCA*-associated cancers involved non-HRR pathway associated CSGs. Overall, non-*BRCA* CSGs represented a greater proportion of PPGVs identified in *BRCA*-associated cancers than *BRCA1/2* (6.9% versus 4.7%). Similarly, non-MMR pathway CSGs represented 11% of PPGVs identified in Lynch Syndrome-associated cancers versus 4.4% of MMR pathway CSGs.

## Discussion

Discovery of inherited cancer predisposition has a myriad of implications. At-risk individuals who have developed cancer can benefit from tailored clinical management including enhanced surveillance strategies and eligibility for precision therapeutics, e.g., PARPi in germline *BRCA*-mutated breast, ovarian, and pancreatic cancer^12–16^. Recognition of hereditary risk may also inspire cascade testing for family members^35^, extending benefit (e.g., more focused screening and consideration of risk-reducing interventions) to others who may be at risk. The ancillary detection via tumor CGP of suspected germline variants that may confer cancer susceptibility is therefore of clinical importance. Guidance for identifying PPGV based on tumor CGP results and assessment of whether follow-up germline genetic testing should be considered has been thoughtfully developed^6,36,37^. To further streamline decision-making for clinicians, a workflow and germline banner reporting feature were implemented to highlight PPGV on FMI tumor CGP results.

Use of a tumor-only sequencing platform necessitated the development of a strategy to refine PPGV calls^6,38–40^ so as to limit unnecessary germline referral and testing and the potential negative consequences thereof, e.g., the economic burden of higher testing volumes on the health care system^41^. In the current study, PPGV calling from tumor CGP involved a number of considerations inclusive of 1) a selected CSG list determined by reported high rates of germline conversion^6^, 2) VAF thresholds designed to capture the majority of true germline variants, and 3) ClinVar pathogenicity classification. When applied to a cohort of > 125,000 cases, our method identified PPGVs in 10.5% of tissue CGP and 6.8% of liquid CGP cases for a combined 9.7% of cases across both assays.

A significant percentage of patients with confirmed pathogenic germline variants identified on tumor CGP do not meet clinical criteria for germline testing, e.g., family and personal cancer history and/or a qualifying diagnosis^3,21–24^. In a study of patients with tumor CGP-identified mutations in moderate risk breast and ovarian CSGs (*ATM, BRIP1, CHEK2, PALB2, RAD51C*, and *RAD51D*), 24% of patients with germline variants would not have met criteria for germline testing^24^. In studies of patients with prostate cancer and with various advanced cancers, 50% and 56% respectively, of patients with actionable variants on confirmatory germline testing following tumor-only sequencing would not have been eligible for germline testing based on current guidelines^21,37^. In our study, while clinical data regarding family history and germline testing eligibility/results were not available precluding a formal analysis of conversion rates, it is notable that the majority of PPGVs (64%, 7771/12,176) were identified in cancer types lacking explicit hereditary testing guidelines.

In particular, tumor CGP can distinctly improve cancer susceptibility determination in the off-tumor setting^2,25,26^. Patients with cancer types not commonly linked to hereditary risk or germline mutations in specific CSGs may be less likely to be evaluated and referred for germline testing than patients diagnosed with classic cancer presentations associated with cancer syndromes. In the current study, a high proportion of select founder mutation PPGVs (30–82%, variable by gene) were identified in tumor types not commonly associated with risk from these genes/mutations yet would be expected to have a high likelihood of germline conversion^36^. The high frequency (47%) of biallelic *MUTYH* founder PPGVs identified in the off-tumor setting is particularly interesting. Given the high penetrance of CRC historically observed in patients with *MUTYH*-Associated Polyposis (MAP) Syndrome, detection of these variants in lung, breast, and other cancers may be suggestive of an expanded phenotype^11^. Indeed, a recent publication exploring a cohort of patients with biallelic *MUTYH* pathogenic germline variants detected through multi-gene panel testing reported phenotypic variability in MAP, e.g., a small population of patients with no personal history of CRC or polyps (8.5%, 7/82) and extracolonic cancers reported in 26% (21/82) of individuals^42^. Whether individual occurrences of biallelic *MUTYH* in our cohort represent causal relationships between *MUTYH*-related base excision repair deficiency (BER) and the diagnosed cancer type versus unveiling unrelated, previously unrecognized hereditary risk is unclear. Additional studies are warranted.

Among cancer types with strong CSG-specific associations and clear germline testing guidance, significant frequencies of PPGVs in other CSGs were observed. Non-HRR CSGs were observed in ~3% of breast, ovarian, pancreatic, and prostate cancers and non-MMR CSGs were observed in ~11% of colorectal and uterine cancers. This suggests that patients who previously tested negative on limited germline panels, such as syndrome-specific assays, may benefit from expanded germline testing prompted by the detection and reporting of a suspicious PPGV on tumor CGP. Even in cancer types for which there is extensive guidance and broad recommendations for germline testing, patients are often unaware of underlying hereditary cancer risk. While nearly all patients with breast, ovarian, pancreatic, and metastatic prostate cancer meet the NCCN criteria for *BRCA1/2* germline testing, one study reported that only 75% of ovarian, 69% of breast, 40% of pancreas, and 18% of patients with prostate cancer had knowledge of their *BRCA1/2* carrier status prior to undergoing tumor CGP^25^.

**Fig. 3:**
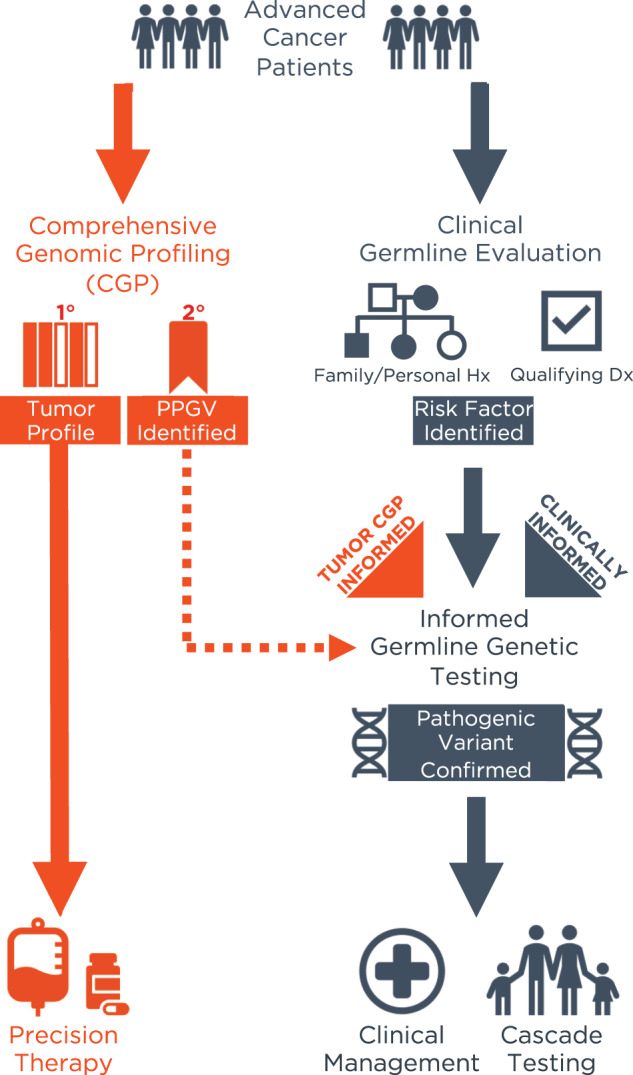
Tumor CGP complements clinically informed germline genetic testing referral. In current practice, referral for germline testing is cancer type-dependent, pursued when clinically indicated due to identification of risk factors upon initial clinical germline evaluation, if performed, and/or in accordance with established guidelines for the tumor type. While the primary purpose of tumor CGP is to inform clinical decision-making regarding cancer treatment, secondary finding of PPGVs can complement clinical germline evaluation in identifying patients who should be referred for germline genetic testing when it may not otherwise be considered with potential implications for clinical management and familial genetic risk assessment. *PPGV* Potential Pathogenic Germline Variant.

Importantly, tumor CGP is not intended to supplant the use of germline genetic testing. Approximately 8% of patients with PPGVs in CSGs may be missed by tumor profiling alone for various reasons, e.g., due to technical differences between assays developed for somatic versus germline interrogation^43^. Definitive identification of a pathogenic germline variant and determination of associated hereditary cancer risk requires dedicated germline testing accompanied by genetic counseling^43–45^. However, barriers to traditional germline genetic testing are well-documented and lead to underutilization of testing and missed/late cancer diagnoses^46,47^. Although some of these barriers, such as racial and socioeconomic disparities^48,49^, may impact both tumor CGP and germline genetic testing, other obstacles – e.g., lengthy wait times for genetic counselors, lack of recognition of clinical criteria for testing by non-genetics specialists, decreased motivation in healthy individuals, and the need for additional clinic visits – are challenges associated with traditional germline genetic testing^46,47^. Thus, the role of tumor CGP should be viewed as complementary to a workflow involving traditional clinical germline evaluation and clinically informed pursuit of germline genetic testing (Fig. 3). By highlighting the identification of potential germline mutations on tumor CGP, we can provide another pathway towards recognizing hereditary cancer risk in families^2,3,22^. Increased awareness of PPGVs among providers can lead to improved screening and prevention strategies for future cancers in patients with a history of cancer as well as for their relatives through cascade testing.

These results should be interpreted in the context of several potential limitations. Implementation of a lower VAF threshold for tissue than for liquid for PPGV filtering may contribute to the difference in the percentage of tissue and liquid PPGV+ cases (10.5% versus 6.8%). The use of separate thresholds for both assays favors sensitivity over specificity and leads to more false positive PPGV calls in tissue. This methodology was motivated by the reduced ability to distinguish germline from somatic origin on tissue CGP compared to liquid CGP in which somatic and germline VAFs are typically separated by 1-2 orders of magnitude^50^. While similar studies separated analyses of hypermutated and non-hypermutated tumors^6^, for the purposes of clinical reporting we applied the same filtering process for identification of PPGVs to all tumors. While PPGVs may be overcalled in MSI-H/TMB-H cases, this impacts a small percentage of patients overall^51,52^. Updated ESMO PMWG guidance recommends inclusion of hypermutated samples for germline focused analysis on tumor CGP given the overall GCR of >5% was maintained for most CSGs in these samples with a disproportionate fraction of true germline variants identified in MMR genes, as expected^27^. Moreover, FMI reports for MSI-H tumors include a recommendation for germline testing for *MLH1*, *MSH2*, *MSH6* and *PMS2* on the basis of the MSI-H biomarker itself (Supplementary Fig. 1D) to rule out the possibility of Lynch Syndrome^36^. The PPGV calling method described here excludes rare germline structural rearrangements from reporting in the germline banner, e.g., *BRCA1/2* large rearrangements most commonly found in individuals of Latin American/Caribbean and/or Middle Eastern descent^53^. However, these rearrangements would still be described and flagged as being of potential germline significance in ‘Potential Germline Implications’ in the Genomic Findings section of the tumor CGP report (Supplementary Fig. 1B, C). In general, the filtering method used here is subject to ancestral bias with the potential for disproportionately undercalling PPGV in minority populations. This is a risk when relying on ClinVar or other large public databases which historically underrepresent minority populations^31–34^. As others have described, the proportion of VUS we identified in predicted non-European populations was markedly higher (e.g., 19.7% in African versus 7.7% in European) and appears to explain much of the disparity in ClinVar-based filtering^33^. While utilization of incomplete public databases risks the propagation of medical disparities for patients from minority populations, collaborative efforts are ongoing to improve and update these resources. The recent announcement from a large diagnostics company declaring their intent to share propriety germline variant classifications with ClinVar gives hope these inequities will be mitigated in future^54^.

Finally, while confirmatory germline testing for this cohort of patients would be instructive to fully capture the impact of the germline banner, we ultimately feel this undertaking is out of scope given our objectives in developing and implementing the methodology herein described. Our approach builds off existing guidance (e.g., ESMO PMWG^6,27^, ACMG^7^) intended to educate regarding recognition of germline potential with the goal of identifying patients who might benefit from germline testing. In developing a composite algorithm (i.e., select CSG list + VAF thresholds + ClinVar evidence), we sought to improve confidence in PPGV calling over other methods (e.g., VAF-based determination). Indeed, in a retrospective study in a population of patients who underwent FMI tumor-only CGP, filtering based on the ESMO-PMWG select CSG list alone improved the GCR from 6.2% to 85.7%^40^, a finding which validates Filter 1 in our combined algorithm and represents the lower limit of improvement on GCR expected in our cohort. While this represents only a partial validation of the germline banner algorithm, we believe this is sufficient as our goal was enrichment for true pathogenic germline variants rather than attainment of specified sensitivity/specificity thresholds.

In a cohort of patients with advanced cancer who underwent either tissue- or liquid-based tumor CGP, a strategy to identify and report PPGV with improved confidence was implemented. Multiple scenarios were identified in which secondary finding of PPGVs via tumor CGP suggested referral for germline testing beyond that indicated by clinical guidelines. Alternative approaches to tumor profiling involve filtering of pathogenic germline variants and reporting practices vary widely among laboratories and institutions. Not reporting this information can result in a missed opportunity to extend the utility of tumor CGP in this manner. Incorporation of both clinical germline evaluation and tumor CGP is crucial to optimize a referral workflow that will yield increased uptake of informed germline genetic testing and lead to improved care and outcomes for patients with cancer and their families.

## Methods

### Study cohort

The study cohort consisted of a research dataset of tumor CGP results reported during routine clinical care between January 2021 and June 2022. Approval for this study, including a waiver of informed consent and a Health Insurance Portability and Accountability Act (HIPAA) waiver of authorization, was obtained from the Western Institutional Review Board (WIRB)-Copernicus Group Institutional Review Board (WCG IRB; Protocol No. 20152817). The WCG IRB granted a waiver of informed consent under 45 CFR § 46.116 based on review and determination that this research meets the following requirements: (i) the research involves no more than minimal risk to the subjects; (ii) the research could not practicably be carried out without the requested waiver; (iii) the waiver will not adversely affect the rights and welfare of the subjects.

### Foundation Medicine comprehensive genomic profiling (CGP)

Hybrid capture-based next-generation sequencing (NGS) assays were performed on patient samples in a Clinical Laboratory Improvement Amendments (CLIA)-certified, College of American Pathologists (CAP)-accredited, New York State-approved laboratory (Foundation Medicine, Inc., Cambridge, MA). FoundationOne^®^CDx and FoundationOne^®^Liquid CDx were performed according to methods previously described^55–57^. Both tissue- and liquid-based testing assessed 324 cancer-related genes and select introns. Microsatellite instability (MSI) status was determined on ≥ 1500 loci^58,59^. Tumor mutational burden (TMB) and blood TMB (bTMB) were determined on a minimum of 0.8 Mb of sequenced DNA per case based on the number of somatic base substitution or short insertion/deletion alterations per Mb after filtering to remove known and likely deleterious somatic mutations and germline single nucleotide polymorphisms (SNPs)^60,61^. The genomic ancestry of patients was determined using a principal component analysis of genomic single nucleotide polymorphisms trained on data from the 1000 Genomes Project with each patient classified as belonging to one of the following subpopulations: African, East Asian, European, South Asian, and Admixed American^62,63^. Detailed variant information for variants discussed in the text and figures is available in Supplementary Table 1.

### Clinicogenomic analysis

Clinical features (e.g., cancer diagnosis, biopsy site, age at biopsy collection) were extracted from test requisition forms and pathology reports. Cases for which features could not be determined were excluded from the analysis. bTMB analysis was restricted to liquid cases with ≥1% ctDNA tumor fraction (cTF), an estimate of circulating tumor DNA (ctDNA) content based on aneuploidy and VAF^64^.

### Statistical analysis

Statistical tests were performed using R (Version 4.2.1) and Python (Version 3.9.12). Fisher’s Exact Tests and Chi-Squared Tests were used, as appropriate, to assess differences between the cohorts and false discovery rate (FDR) corrections were made using the p.adjust function in R to correct P values for multiple tests. Fisher’s Exact Tests were also carried out using the “fisher_exact” function from the statistical functions module (scipy.stats) of Scipy (v1.7.3) and corrections for multiple hypothesis tests were performed using the Benjamini-Hochberg Procedure with the “fdr_bh” method for the “multipletests” function from the statsmodels (v0.13.2) Python package.

### Reporting summary

Further information on research design is available in the Nature Research Reporting Summary linked to this article.

### Supplementary information

Supplementary Information

Reporting Summary

## Data Availability

The authors declare that all relevant aggregate data supporting the findings of this study are available within the article and its supplementary information files. In accordance with the Health Insurance Portability and Accountability Act, we do not have IRB approval or patient consent to share individualized patient genomic data, which contains potentially identifying or sensitive patient information and cannot be reported in a public data repository. Foundation Medicine is committed to collaborative data analysis and has well established and widely used mechanisms by which qualified researchers can query our core genomic database of > 700,000 de-identified sequenced cancers. Academic researchers can submit a proposal to the Foundation Medicine Data Collaborations Committee and, if approved, the researcher/institution will be required to complete a Data Usage Agreement. More information and mechanisms for data access can be obtained by contacting the corresponding author or the Foundation Medicine Data Governance Council at data.governance.council@foundationmedicine.com.

## Code availability

This study made use of publicly available packages with Python 3.9.12 and R version 4.2.1. Code used to generate figures is available upon request.
