# Supplementary material for: Potential pathogenic germline variant reporting from tumor comprehensive genomic profiling complements classic approaches to germline testing
Source: NPJ Precis Oncol. 2023 Aug 11;7:76. doi: 10.1038/s41698-023-00429-1 (PMC10421918; doi:10.1038/s41698-023-00429-1)
Supplement: Supplementary file 1 — Supplementary Information [file 41698_2023_429_MOESM1_ESM.pdf]

## Supplementary Figure 1. Foundation Medicine Reporting Of Secondary Germline Findings

**a**

GERMLINE BANNER

**VARIANTS TO CONSIDER FOR FOLLOW-UP GERMLINE TESTING IN SELECT CANCER SUSCEPTIBILITY GENES**

*Findings below have been previously reported as pathogenic germline in the ClinVar genomic database and were detected at an allele frequency of >10%. See appendix for details.*

***BRCA2*** **-** S1982fs*22 ........ p. 4

This report does not indicate whether variants listed above are germline or somatic in this patient. In the appropriate clinical context, follow-up germline testing would be needed to determine whether a finding is germline or somatic.

**b**

PPGV W/ CLINVAR EVIDENCE

**POTENTIAL GERMLINE IMPLICATIONS**

The alteration seen here is one of several BRCA1/2 founder mutations seen with disproportionately high frequency in the Ashkenazi Jewish population, the most common mutations being BRCA1 185delAG (E23fs*17), BRCA1 5382insC (Q1756fs*74), and BRCA2 6174delT (S1982fs*22)[111]. These variants have been described in the ClinVar database as pathogenic germline mutations associated with hereditary breast and ovarian cancer syndrome (ClinVar, Sep 2022)[112]. Follow-up germline testing would be needed to distinguish whether the finding in this patient is somatic or germline. Inactivating germline mutations in BRCA1 or BRCA2 are associated with autosomal dominant hereditary breast and ovarian cancer[113-114], and the lifetime risk of breast and ovarian cancer in BRCA2 mutation carriers has been estimated to be as high as >80% and 23%, respectively[115]. Elevated risk for other cancer types, including gastric, pancreatic, prostate, and colorectal, has also been identified, with an increase in risk ranging from 20 to 60%[116]. The estimated prevalence of deleterious germline BRCA1/2 mutations in the general population is between 1:400 and 1:800, with an approximately 10-fold higher prevalence in the Ashkenazi Jewish population[115,117-122]. In the appropriate clinical context, germline testing of BRCA2 is recommended.

**c**

PPGV W/O CLINVAR EVIDENCE

**POTENTIAL GERMLINE IMPLICATIONS**

Inactivating germline mutations in BRCA1 or BRCA2 are associated with autosomal dominant hereditary breast and ovarian cancer[89-90], and the lifetime risk of breast and ovarian cancer in BRCA1/2 mutation carriers has been estimated to be as high as 87% and 44%, respectively[91]. Elevated risk for other cancer types, including gastric, pancreatic, prostate, and colorectal, has also been identified, with an increase in risk ranging from 20 to 60%[92]. The estimated prevalence of deleterious germline BRCA1/2 mutations in the general population is between 1:400 and 1:800, with an approximately 10-fold higher prevalence in the Ashkenazi Jewish population[91,93-98]. In the appropriate clinical context, germline testing of BRCA1 is recommended.

**d**

MSI-H BIOMARKER

**POTENTIAL GERMLINE IMPLICATIONS**

While approximately 80% of MSI-H tumors arise due to somatic inactivation of an MMR pathway protein, about 20% arise due to germline mutations in one of the MMR genes[21], which are associated with a condition known as Lynch syndrome (also known as hereditary nonpolyposis colorectal cancer or HNPCC)[34]. Lynch syndrome leads to an increased risk of colorectal, endometrial, gastric, and other cancers[34-36] and has an estimated prevalence in the general population ranging from 1:600 to 1:2000[37-39]. Therefore, in the appropriate clinical context, germline testing of MLH1, MSH2, MSH6, and PMS2 is recommended.

Example Foundation Medicine reporting of secondary germline findings: a) Germline Banner report feature located on Page 1 of Professional Services; b) ‘Potential Germline Implications’ discussion in Germline Findings section for PPGV reported in ClinVar; c) ‘Potential Germline Implications’ discussion in Germline Findings section for PPGV not reported in ClinVar; and d) ‘Potential Germline Implications’ discussion in Biomarker Findings section based on detection of MSI-H biomarker. MSI-H, Microsatellite Instability-High; PPGV, Potential Pathogenic Germline Variants.

# Supplementary Figure 2. Development & Validation Of PPGV VAF Thresholds

**a** Exemplar Germline Variants (EGV)

| Gene | Coding Sequence Effect | Protein Sequence Effect |
|---|---|---|
| *BRCA1* | 114G>A | K38K |
| *BRCA1* | 591C>T | C197C |
| *BRCA1* | 1067A>G | Q356R |
| *BRCA1* | 1971A>G | Q657Q |
| *BRCA1* | 2077G>A | D693N |
| *BRCA1* | 2458A>G | K820E |
| *BRCA1* | 2521C>T | R841W |
| *BRCA1* | 3119G>A | S1040N |
| *BRCA1* | 3418A>G | S1140G |
| *BRCA1* | 4039A>G | R1347G |
| *BRCA1* | 4535G>T | S1512I |
| *BRCA1* | 4956G>A | M1652I |
| *BRCA2* | 125A>G | Y42C |
| *BRCA2* | 865A>C | N289H |
| *BRCA2* | 1365A>G | S455S |
| *BRCA2* | 1788T>C | D596D |
| *BRCA2* | 2229T>C | H743H |
| *BRCA2* | 2971A>G | N991D |
| *BRCA2* | 3264T>C | P1088P |
| *BRCA2* | 3516G>A | S1172S |
| *BRCA2* | 4068G>A | L1356L |
| *BRCA2* | 4090A>C | I1364L |
| *BRCA2* | 4258G>T | D1420Y |
| *BRCA2* | 5199C>T | S1733S |
| *BRCA2* | 5418A>G | E1806E |
| *BRCA2* | 5704G>A | D1902N |
| *BRCA2* | 5744C>T | T1915M |
| *BRCA2* | 6100C>T | R2034C |
| *BRCA2* | 6347A>G | H2116R |
| *BRCA2* | 7017G>C | K2339N |
| *BRCA2* | 7319A>G | H2440R |
| *BRCA2* | 7469T>C | I2490T |
| *BRCA2* | 8149G>T | A2717S |
| *BRCA2* | 8182G>A | V2728I |
| *BRCA2* | 8460A>C | V2820V |
| *BRCA2* | 8567A>C | E2856A |
| *BRCA2* | 8830A>T | I2944F |
| *BRCA2* | 8851G>A | A2951T |
| *BRCA2* | 9730G>A | V3244I |
| *BRCA2* | 9976A>T | K3326* |
| *BRCA2* | 10110G>A | R3370R |
| *BRCA2* | 10234A>G | I3412V |
| *TP53* | 108G>A | P36P |
| *TP53* | 139C>T | P47S |
| *TP53* | 639A>G | R213R |

**b**

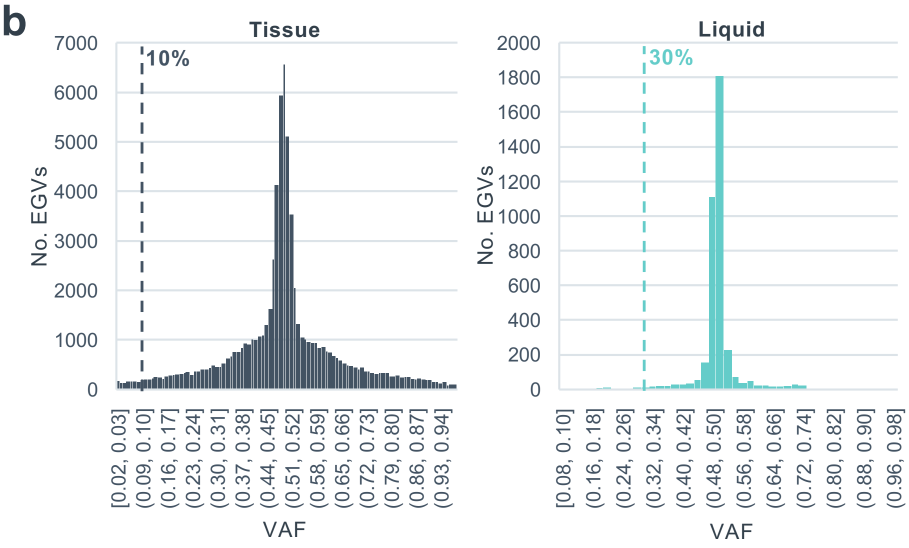


**c** VAF Threshold Validation

| Test Type | VAF Threshold | Cancer Type | % EGV w/VAF Above Threshold | No. Of Variants |
|---|---|---|---|---|
| F1CDx® / FoundationOne® | >10% VAF | Pan-Cancer | 98.2% | 82,162 |
| | | Lung | 98.6% | 13,535 |
| | | Breast | 98.2% | 8,055 |
| | | Prostate | 98.2% | 2,510 |
| | | Ovary | 96.6% | 4,728 |
| | | Glioma | 97.7% | 2,565 |
| F1LCDx™ / FoundationOne®Liquid | >30% VAF | Pan-Cancer | 98.1% | 3,969 |
| | | Lung | 98.7% | 1,360 |
| | | Breast | 98.7% | 393 |
| | | Prostate | 100.0% | 284 |
| | | Ovary | 100.0% | 79 |
| | | Glioma | 100.0% | 19 |

a) The VAF distribution of a set of exemplar germline variants (EGV) was plotted in both tissue and liquid CGP samples with the goal of identifying a threshold below which germline variants are unlikely to be present on tumor CGP. A set of common variants was chosen for this analysis as they would be expected to occur rarely as somatic variants in cancer. Variants in *BRCA1*, *BRCA2*, and *TP53* were chosen since these genes are frequently under loss of heterozygosity (LOH) in tumors. Variants with high population allele frequencies were excluded as they are frequently homozygous. Variants with VAF <2% and >98% were also excluded as they are unlikely to be heterozygous germline in a given sample. b) Based on this analysis, a threshold of >10% VAF for tissue CGP (FoundationOne®CDx, FoundationOne®) and >30% VAF for liquid CGP (FoundationOne®Liquid CDx, FoundationOne®Liquid) was selected. c) Applying these thresholds, there is high confidence that >95% of germline variants are identified across tumor CGP assays and across cancer types. CGP, Comprehensive Genomic Profiling; F1CDx®, FoundationOne®CDx; F1LCDx™, FoundationOne®Liquid CDx; VAF, Variant Allele Frequency.

# Supplementary Figure 3. Cancer Types Profiled Using Tissue & Liquid Tumor CGP

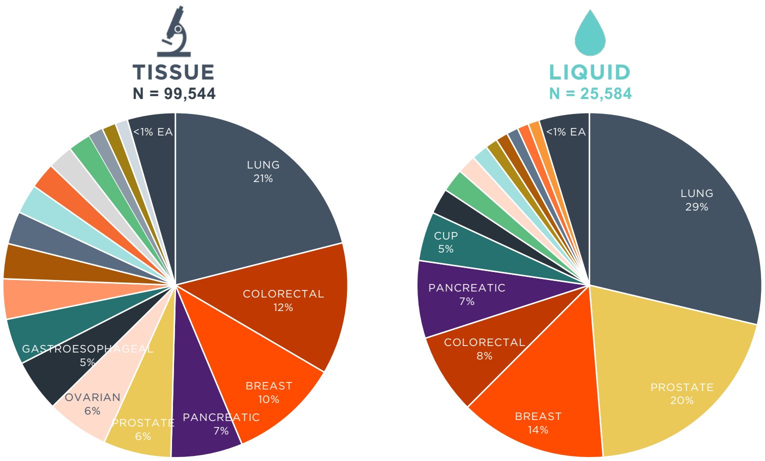


| | |
|---|---|
| CUP | 4% |
| UTERINE | 4% |
| UROTHELIAL | 3% |
| MELANOMA | 3% |
| NEUROENDOCRINE | 3% |
| HEAD & NECK | 2% |
| CNS/BRAIN | 2% |
| CHOLANGIOCARCINOMA | 2% |
| GI OTHER | 1% |
| NON-UROTHELIAL KIDNEY | 1% |
| OTHER | 1% |
| CERVICAL | <1% |
| THYROID | <1% |
| SOFT TISSUE SARCOMA | <1% |
| GALLBLADDER | <1% |
| LIVER | <1% |
| NON-MELANOMA SKIN | <1% |
| GERM CELL | <1% |
| HEMATOLOGIC | <1% |

| | |
|---|---|
| GASTROESOPHAGEAL | 2% |
| CHOLANGIOCARCINOMA | 2% |
| OVARIAN | 2% |
| NEUROENDOCRINE | 2% |
| NON-UROTHELIAL KIDNEY | 1% |
| UROTHELIAL | 1% |
| MELANOMA | 1% |
| HEAD & NECK | 1% |
| SOFT TISSUE SARCOMA | 1% |
| UTERINE | <1% |
| GI OTHER | <1% |
| THYROID | <1% |
| HEMATOLOGIC | <1% |
| GALLBLADDER | <1% |
| OTHER | <1% |
| LIVER | <1% |
| CERVICAL | <1% |
| CNS/BRAIN | <1% |
| NON-MELANOMA SKIN | <1% |
| GERM CELL | <1% |

Percentages reflect the % of total tissue or liquid cases profiled, respectively.

**Supplementary Figure 4. Multi-PPGV Samples Are Partially Explained By Underlying MSI-H/TMB-H**

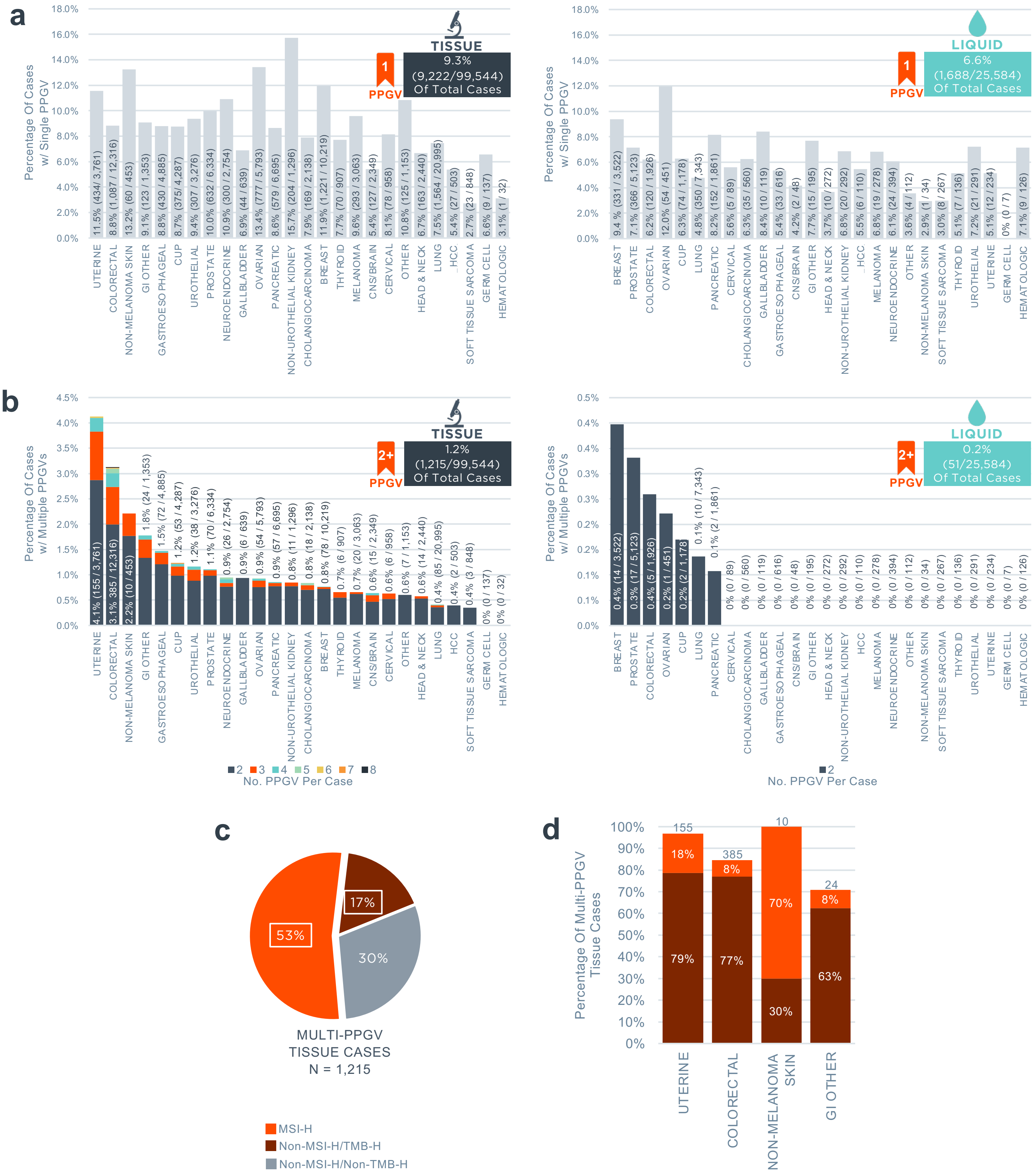


a) Percentage of cases in tissue (*Left*) and liquid (*Right*) CGP cohorts in which a single PPGV was identified. b) Percentage of cases in tissue (*Left*) and liquid (*Right*) CGP in which multiple PPGVs were identified. c) MSI and TMB status of the 1,215 tissue CGP cases with multiple PPGV. TMB-H was defined as ≥10 Mut/Mb. d) The MSI-H and Non-MSI-H/TMB-H breakdown for cancer types with the highest percentage (>1.5%) of cases with multiple PPGVs are shown. MSI-H, Microsatellite Instability-High; PPGV, Potential Pathogenic Germline Variant; TMB-H, Tumor Mutational Burden-High.

## Supplementary Figure 5. Filtering Of Liquid Tumor CGP Variants On The Basis Of ClinVar Classification

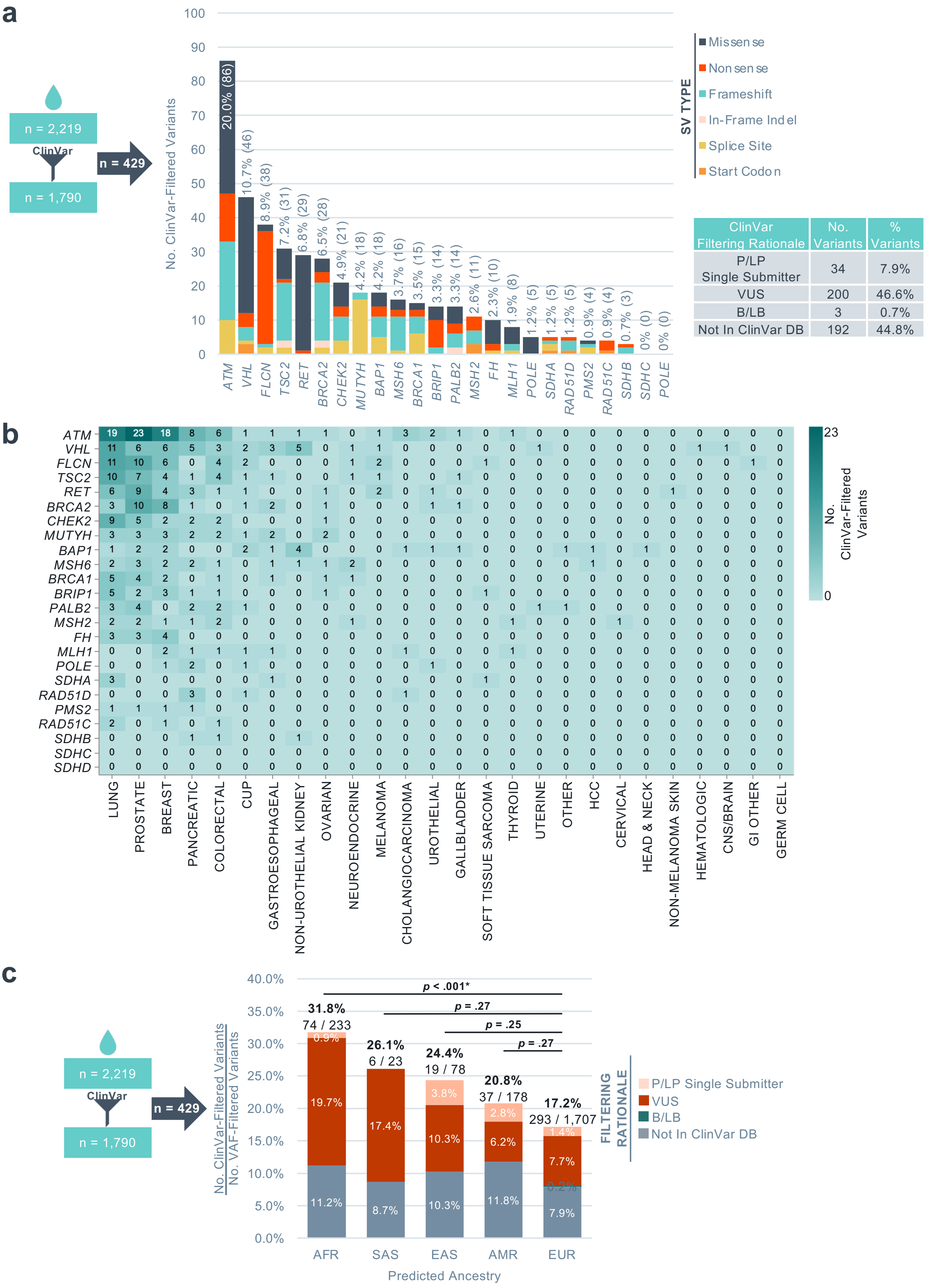


| ClinVar Filtering Rationale | No. Variants | % Variants |
|---|---|---|
| P/LP Single Submitter | 34 | 7.9% |
| VUS | 200 | 46.6% |
| B/LB | 3 | 0.7% |
| Not In ClinVar DB | 192 | 44.8% |

a) Pan-cancer distribution of ClinVar-filtered variants from liquid tumor CGP cases across 24 germline banner CSGs and rationale for filtering out (Table). 429 SV across 422 cases were excluded. b) Pan-tumor landscape of ClinVar-filtered variants from liquid CGP cases in 24 germline banner genes. Values in the heatmap reflect the number of filtered variants. The x-axis is arranged by the total number of filtered variants per cancer type. The y-axis is arranged by the total number of filtered variants per CSG. c) Association of predicted patient ancestry and ClinVar-filtered variants. Percentages reflect the ratio of variants removed after the ClinVar filtering step to variants retained after the VAF filtering step, i.e., the ratio of ClinVar-filtered to pre-ClinVar-filtered variants for each ancestry. Statistical analysis was performed using Fisher's Exact Test with the Benjamini-Hochberg Procedure for p-value multiple hypothesis corrections. AFR, African; AMR, Admixed American; B/LB, Benign/Likely Benign; CGP, Comprehensive Genomic Profiling; CSG, Cancer Susceptibility Gene; DB, Database; EAS, East Asian; EUR, European; P/LP, Pathogenic/Likely Pathogenic; PPGV, Potential Pathogenic Germline Variant; SAS, South Asian; SV, Short Variant; VUS, Variant Of Uncertain Significance.

## Supplementary Figure 6. Pan-Tumor Landscape Of PPGVs Across 24 Germline Banner CSGs (Tissue & Liquid)

**a**

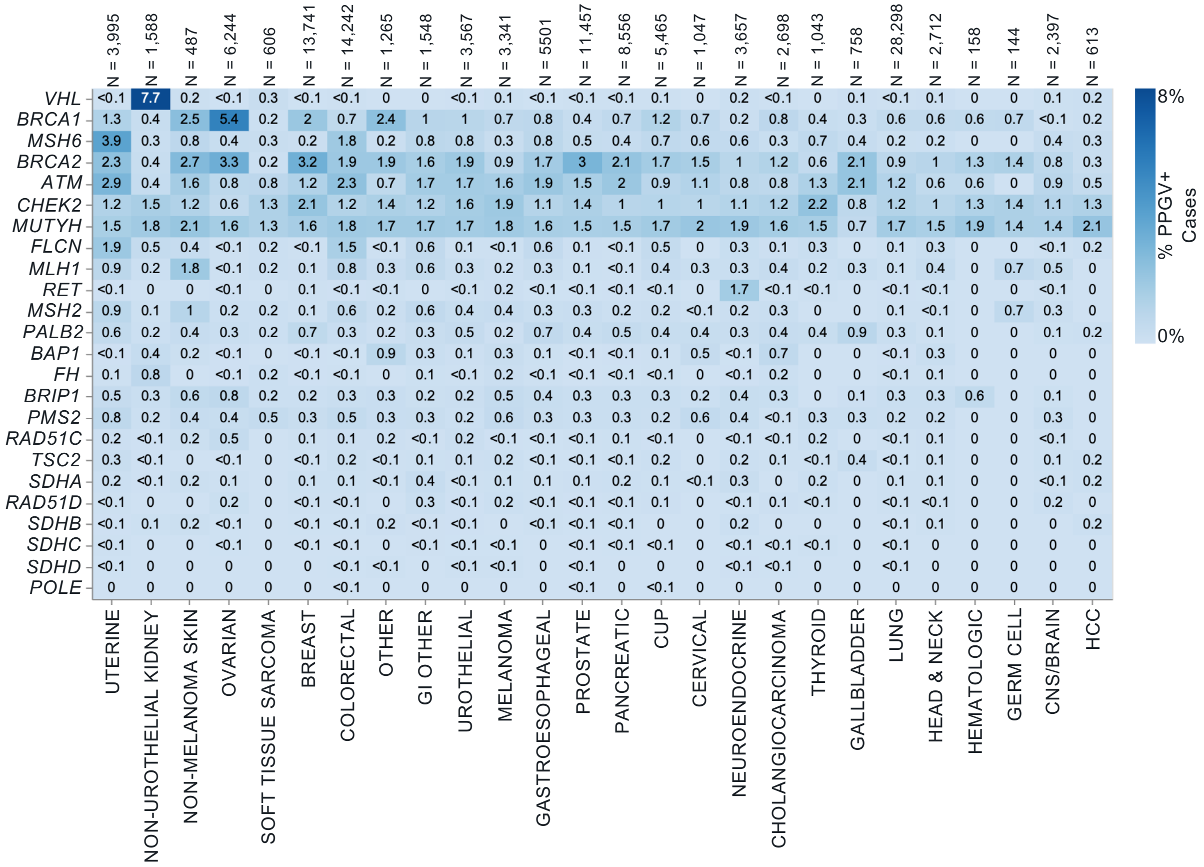

## Supplementary Figure 6. Pan-Tumor Landscape Of PPGVs Across 24 Germline Banner CSGs (Tissue & Liquid)

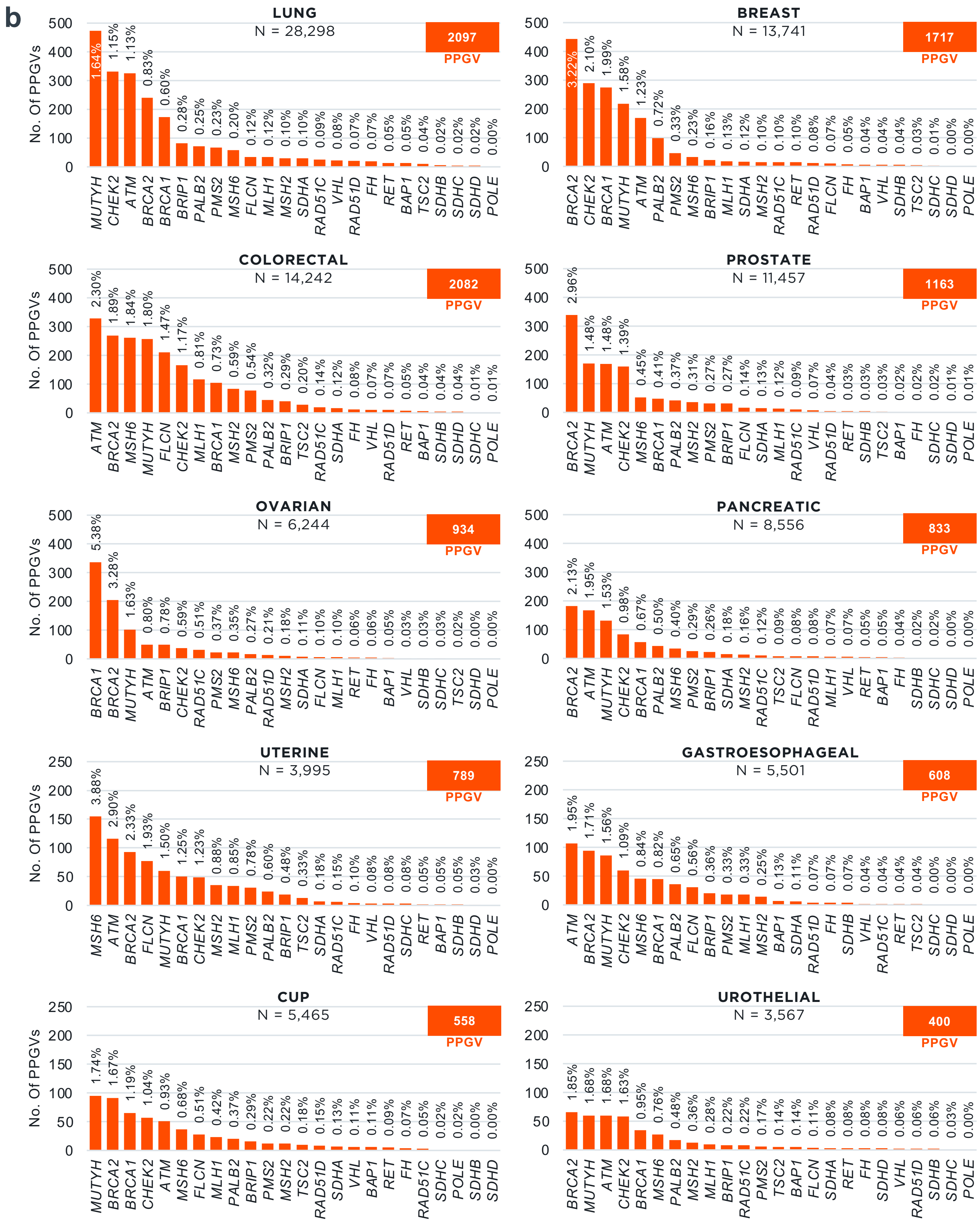

## Supplementary Figure 6. Pan-Tumor Landscape Of PPGVs Across 24 Germline Banner CSGs (Tissue & Liquid)

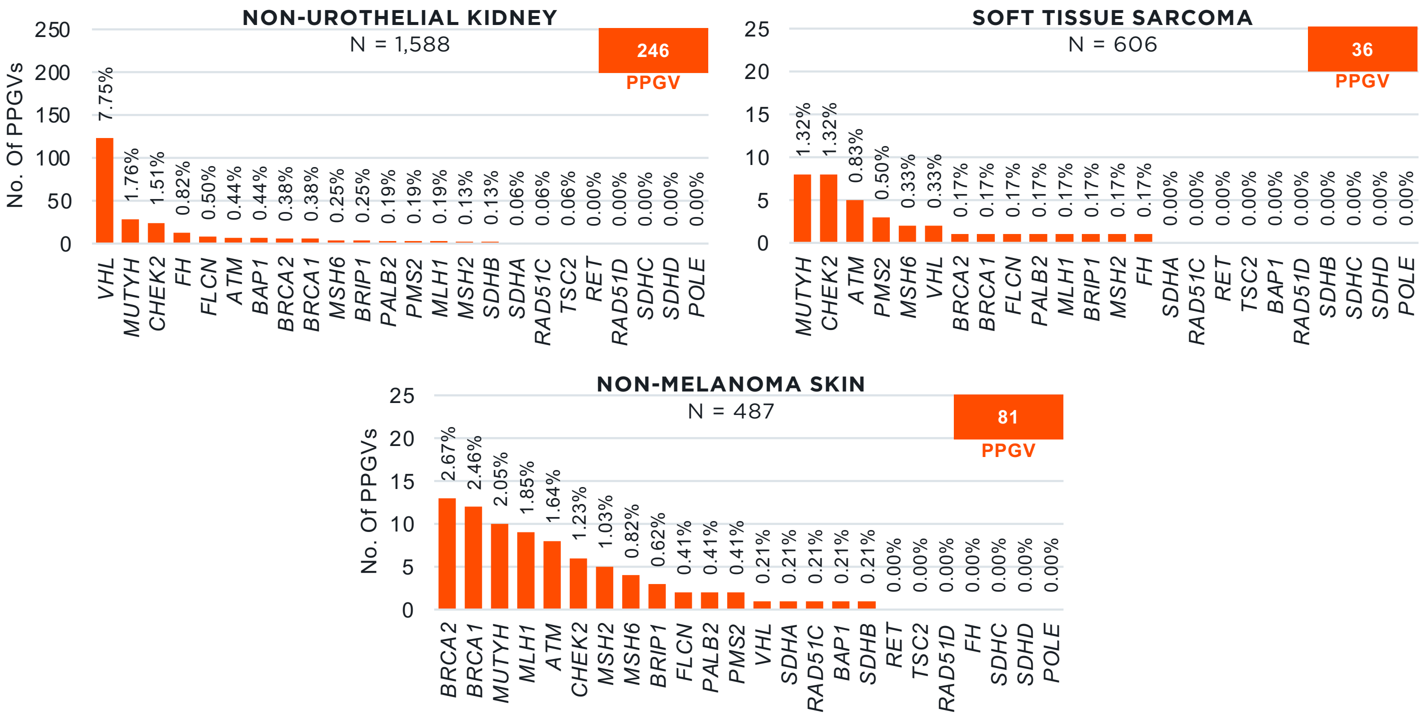


a) Values in the heatmap reflect the % of total cases per cancer type (N) with PPGVs detected in combined tissue and liquid CGP. The x-axis is arranged by the overall % of PPGV+ cases per cancer type (refer to **Figure 2A**). The y-axis is arranged according to the maximum cancer type-specific prevalence for each gene. b) Long tail plots showing the distribution of PPGVs across the 24 germline banner CSGs in cancer types/groupings with either PPGV prevalence ≥10% and/or PPGV incidence ≥500 samples (n = 13). Percentages represent gene-specific PPGV prevalence in the given cancer type. CSG, Cancer Susceptibility Gene; PPGV, Potential Pathogenic Germline Variant.

## Supplementary Figure 7. 'Off-Tumor' Cancer Types With Founder Mutation PPGVs (Related To Figure 2B)

**a**

*BRCA1*
E23fs*17, C61G, Q1756fs*74

| 'OFF-TUMOR' TYPE | NO. CASES | % CASES |
|---|---|---|
| LUNG | 21 | 11.4% |
| CUP | 14 | 7.6% |
| GASTROESOPHAGEAL | 11 | 6.0% |
| UROTHELIAL | 8 | 4.3% |
| OTHER | 6 | 3.3% |
| COLORECTAL | 6 | 3.3% |
| CHOLANGIOCARCINOMA | 5 | 2.7% |
| UTERINE | 5 | 2.7% |
| NON-UROTHELIAL KIDNEY | 2 | 1.1% |
| MELANOMA | 2 | 1.1% |
| HCC | 1 | 0.5% |
| CNS/BRAIN | 1 | 0.5% |
| NEUROENDOCRINE | 1 | 0.5% |
| HEAD & NECK | 1 | 0.5% |

**b**

*BRCA2*
E1308*, S1982fs*22

| 'OFF-TUMOR' TYPE | NO. CASES | % CASES |
|---|---|---|
| LUNG | 7 | 5.6% |
| COLORECTAL | 7 | 5.6% |
| GASTROESOPHAGEAL | 6 | 4.8% |
| UTERINE | 3 | 2.4% |
| CUP | 3 | 2.4% |
| HEMATOLOGIC | 2 | 1.6% |
| GI OTHER | 2 | 1.6% |
| UROTHELIAL | 2 | 1.6% |
| OTHER | 2 | 1.6% |
| HEAD & NECK | 2 | 1.6% |
| MELANOMA | 1 | 0.8% |
| CERVICAL | 1 | 0.8% |

**c**

*ATM*
V2424G

| 'OFF-TUMOR' TYPE | NO. CASES | % CASES |
|---|---|---|
| LUNG | 18 | 41.9% |
| PROSTATE | 4 | 9.3% |
| COLORECTAL | 4 | 9.3% |
| OTHER | 2 | 4.7% |
| GASTROESOPHAGEAL | 2 | 4.7% |
| CUP | 2 | 4.7% |
| MELANOMA | 1 | 2.3% |
| UROTHELIAL | 1 | 2.3% |
| CHOLANGIOCARCINOMA | 1 | 2.3% |

**d**

*CHEK2*
T367fs*15

| 'OFF-TUMOR' TYPE | NO. CASES | % CASES |
|---|---|---|
| LUNG | 108 | 19.8% |
| PROSTATE | 66 | 12.1% |
| CUP | 25 | 4.6% |
| MELANOMA | 20 | 3.7% |
| GASTROESOPHAGEAL | 17 | 3.1% |
| PANCREATIC | 17 | 3.1% |
| UROTHELIAL | 16 | 2.9% |
| NON-UROTHELIAL KIDNEY | 13 | 2.4% |
| UTERINE | 12 | 2.2% |
| OVARIAN | 10 | 1.8% |
| CHOLANGIOCARCINOMA | 9 | 1.7% |
| NEUROENDOCRINE | 9 | 1.7% |
| THYROID | 8 | 1.5% |
| CNS/BRAIN | 7 | 1.3% |
| GI OTHER | 5 | 0.9% |
| CERVICAL | 4 | 0.7% |
| HEAD & NECK | 4 | 0.7% |
| OTHER | 4 | 0.7% |
| SOFT TISSUE SARCOMA | 2 | 0.4% |
| GALLBLADDER | 1 | 0.2% |
| HEMATOLOGIC | 1 | 0.2% |
| HCC | 1 | 0.2% |
| NON-MELANOMA SKIN | 1 | 0.2% |

**e**

*MUTYH* Biallelic
Y165C, G382D

| 'OFF-TUMOR' TYPE | NO. CASES | % CASES |
|---|---|---|
| LUNG | 5 | 13.2% |
| BREAST | 4 | 10.5% |
| PROSTATE | 3 | 7.9% |
| GI OTHER | 2 | 5.3% |
| MELANOMA | 1 | 2.6% |
| PANCREAS | 1 | 2.6% |
| NEUROENDOCRINE | 1 | 2.6% |
| CUP | 1 | 2.6% |

COLORECTAL (n = 20)

| BIALLELIC *MUTYH* PPGV | NO. CASES | % CASES |
|---|---|---|
| Y165C/G382D | 6 | 30% |
| G382D/G382D | 5 | 25% |
| Y165C/OTHER | 4 | 20% |
| G382D/OTHER | 4 | 20% |
| Y165C/Y165C | 1 | 5% |
| TOTAL | 20 | 100% |

NON-COLORECTAL (n = 18)

| BIALLELIC *MUTYH* PPGV | NO. CASES | % CASES |
|---|---|---|
| G382D/G382D | 7 | 39% |
| Y165C/G382D | 6 | 33% |
| G382D/OTHER | 3 | 17% |
| Y165C/Y165C | 1 | 6% |
| Y165C/OTHER | 1 | 6% |
| TOTAL | 18 | 100% |

Percentages reflect the % of total cases harboring the indicated founder mutation(s) across all cancer types in combined tissue and liquid CGP. For biallelic *MUTYH* PPGV, homozygous versus compound heterozygous status for colorectal versus non-colorectal cases is provided.

## Supplementary Table 1. Variants Highlighted In Text & Figures

| GENE | NCBI REFERENCE SEQUENCE | CODING SEQUENCE EFFECT | PROTEIN SEQUENCE EFFECT |
|---|---|---|---|
| *ATM* | NM_000051 | c.7271T>G | p.V2424G |
| *BRCA1* | NM_007294 | c.68_69delAG | p.E23fs*17 |
| *BRCA1* | NM_007294 | c.181T>G | p.C61G |
| *BRCA1* | NM_007294 | c.971delG | p.S324fs*17 |
| *BRCA1* | NM_007294 | c.5266_5267insC | p.Q1756fs*74 |
| *BRCA2* | NM_000059 | c.2937delA | p.I979fs*12 |
| *BRCA2* | NM_000059 | c.3922G>T | p.E1308* |
| *BRCA2* | NM_000059 | c.5946delT | p.S1982fs*22 |
| *CHEK2* | NM_007194 | c.1100delC | p.T367fs*15 |
| *MUTYH* | NM_001048171 | c.494A>G | p.Y165C |
| *MUTYH* | NM_001048171 | c.1145G>A | p.G382D |
