# Supplementary material for: Potential pathogenic germline variant reporting from tumor comprehensive genomic profiling complements classic approaches to germline testing
Source: NPJ Precis Oncol. 2023 Aug 11;7:76. doi: 10.1038/s41698-023-00429-1 (PMC10421918; doi:10.1038/s41698-023-00429-1)
Supplement: Supplementary file 2 — Reporting Summary [file 41698_2023_429_MOESM2_ESM.pdf]

nature portfolio

Corresponding author(s): Rachel Beth Keller

Last updated by author(s): Jul 10, 2023

Reporting Summary

Nature Portfolio wishes to improve the reproducibility of the work that we publish. This form provides structure for consistency and transparency in reporting. For further information on Nature Portfolio policies, see our Editorial Policies and the Editorial Policy Checklist.

Statistics

For all statistical analyses, confirm that the following items are present in the figure legend, table legend, main text, or Methods section.

n/a	Confirmed	
☐	☒	The exact sample size (n) for each experimental group/condition, given as a discrete number and unit of measurement
☐	☒	A statement on whether measurements were taken from distinct samples or whether the same sample was measured repeatedly
☐	☒	The statistical test(s) used AND whether they are one- or two-sided<br>*Only common tests should be described solely by name; describe more complex techniques in the Methods section.*
☐	☒	A description of all covariates tested
☐	☒	A description of any assumptions or corrections, such as tests of normality and adjustment for multiple comparisons
☐	☒	A full description of the statistical parameters including central tendency (e.g. means) or other basic estimates (e.g. regression coefficient) AND variation (e.g. standard deviation) or associated estimates of uncertainty (e.g. confidence intervals)
☐	☒	For null hypothesis testing, the test statistic (e.g. F, t, r) with confidence intervals, effect sizes, degrees of freedom and P value noted<br>*Give P values as exact values whenever suitable.*
☒	☐	For Bayesian analysis, information on the choice of priors and Markov chain Monte Carlo settings
☒	☐	For hierarchical and complex designs, identification of the appropriate level for tests and full reporting of outcomes
☒	☐	Estimates of effect sizes (e.g. Cohen's d, Pearson's r), indicating how they were calculated

Our web collection on statistics for biologists contains articles on many of the points above.

Software and code

Policy information about availability of computer code

Data collection	No software was used for data collection and no data collection was performed for this study. This study utilized the Foundation Medicine genomic database (FoundationCore®).
Data analysis	Custom scripts using Python 3.9.12 were used for data analysis and figure generation.

For manuscripts utilizing custom algorithms or software that are central to the research but not yet described in published literature, software must be made available to editors and reviewers. We strongly encourage code deposition in a community repository (e.g. GitHub). See the Nature Portfolio guidelines for submitting code & software for further information.

Data

Policy information about availability of data

All manuscripts must include a data availability statement. This statement should provide the following information, where applicable:

- Accession codes, unique identifiers, or web links for publicly available datasets
- A description of any restrictions on data availability
- For clinical datasets or third party data, please ensure that the statement adheres to our policy

The authors declare that all relevant aggregate data supporting the findings of this study are available within the article and its supplementary information files. In accordance with the Health Insurance Portability and Accountability Act, we do not

have IRB approval or patient consent to share individualized patient genomic data, which contains potentially identifying or sensitive patient information and cannot be reported in a public data repository. Foundation Medicine is committed to collaborative data analysis and has well established and widely used mechanisms by which qualified researchers can query our core genomic database of >700,000 de-identified sequenced cancers. More information and mechanisms for data access can be obtained by contacting the corresponding author or the Foundation Medicine Data Governance Council at data.governance.council@ffoooundationmedicine.com.

Human research participants

Policy information about studies involving human research participants and Sex and Gender in Research.

Reporting on sex and gender	Patient sex was abstracted from the Foundation Medicine (FMI) order form and summarized for the pan-tumor cohort in Table 1.
Population characteristics	FoundationCore® includes patients with cancer whose tumor samples were submitted for comprehensive genomic profiling (CGP) during routine clinical care. The majority of cases analyzed were adults (>18 years of age) with advanced cancer, and a significant fraction of these patients are likely deceased. This is retrospective research that involves no more than a minimal risk to the privacy of patients and involves no intervention or contact with patients. FMI provides CGP test results at the request of treating physicians and therefore has no direct relationship with any of these patients. Moreover, in many cases the patients may no longer be associated with the treating physician who ordered their FMI test or may be deceased, and therefore it may be impossible to contact these patients.
Recruitment	All patients included in FoundationCore® had tumor samples sent by their treating physician for CGP during routine clinical care. This was a retrospective analysis of data which already existed in the database.
Ethics oversight	For FoundationCore® analysis, approval for this study, including a waiver of informed consent and a HIPAA waiver of authorization, was obtained from the WCG Institutional Review Board (IRB; Protocol No. 20152817).

Note that full information on the approval of the study protocol must also be provided in the manuscript.

Field-specific reporting

Please select the one below that is the best fit for your research. If you are not sure, read the appropriate sections before making your selection.

☒ Life sciences ☐ Behavioural & social sciences ☐ Ecological, evolutionary & environmental sciences

For a reference copy of the document with all sections, see nature.com/documents/nr-reporting-summary-flat.pdf

Life sciences study design

All studies must disclose on these points even when the disclosure is negative.

Sample size	This was a retrospective study utilizing FoundationCore® data from cases submitted for CGP during routine clinical care. As such, no methods were used to predetermine sample size (n).
Data exclusions	Duplicate patient samples in FoundationCore® were excluded from the cohort so as to limit the analysis to one unique sample per patient.
Replication	All analysis in this study was descriptive, not experimental, therefore this design consideration does not apply.
Randomization	All analysis in this study was descriptive, not experimental, therefore this design consideration does not apply.
Blinding	All analysis in this study was descriptive, not experimental, therefore this design consideration does not apply.

Reporting for specific materials, systems and methods

We require information from authors about some types of materials, experimental systems and methods used in many studies. Here, indicate whether each material, system or method listed is relevant to your study. If you are not sure if a list item applies to your research, read the appropriate section before selecting a response.

Materials & experimental systems

n/a	Involved in the study
☒	☐ Antibodies
☒	☐ Eukaryotic cell lines
☒	☐ Palaeontology and archaeology
☒	☐ Animals and other organisms
☒	☐ Clinical data
☒	☐ Dual use research of concern

Methods

n/a	Involved in the study
☒	☐ ChIP-seq
☒	☐ Flow cytometry
☒	☐ MRI-based neuroimaging
